# Mesenteric inflammatory veno-occlusive disease occurring during the course of ulcerative colitis: a case report

**DOI:** 10.1186/s12876-018-0737-7

**Published:** 2018-01-11

**Authors:** Yosuke Yamada, Ken Sugimoto, Yashiro Yoshizawa, Yoshifumi Arai, Yoshiro Otsuki, Tomio Arai, Yasuyuki Kobayashi, Yoshihiko Sato, Yoshisuke Hosoda

**Affiliations:** 1 Department of Gastroenterology, Seirei Hamamatsu General Hospital, Hamamatsu, 430-8558 Japan; 2 First Department of Medicine, Hamamatsu University School of Medicine, 1-20-1 Handayama, Hamamatsu, 431-3192 Japan; 3 Department of Pathology, Seirei Hamamatsu General Hospital, Hamamatsu, 430-8558 Japan; 4 Department of Pathology, Tokyo Metropolitan Geriatric Hospital and Institute of Gerontology, Tokyo, 173-0015 Japan; 5 Department of Surgery, Seirei Hamamatsu General Hospital, Hamamatsu, 430-8558 Japan

**Keywords:** Mesenteric inflammatory veno-occlusive disease, Enterocolic lymphocytic phlebitis, Intramural mesenteric venulitis, Chronic intestinal lymphocytic micro phlebitis, Ulcerative colitis

## Abstract

**Background:**

Mesenteric inflammatory veno-occlusive disease (MIVOD) is difficult to diagnose because of its rarity, nonspecific clinical findings, and frequent confusion with other diseases including inflammatory bowel disease. This report presents a very rare case of MIVOD that occurred during the course of ulcerative colitis (UC).

**Case presentation:**

A 32-year-old man, who had been diagnosed with UC at the age of 29 and was in remission maintained by oral administration of 5-aminosalicylic acid (5-ASA), showed exacerbation of diarrhea and was admitted to the hospital. Since it was deemed an exacerbation of UC, intravenous steroid therapy and oral administration of tacrolimus were initiated, but his condition continued to worsen. Abdominal computed tomography (CT) was performed and showed intraperitoneal free air, leading to a diagnosis of gastrointestinal perforation and the performance of emergency surgery (subtotal colectomy and ileostomy). Histopathological examination of the resected colon of the patient showed mucosal inflammatory findings that were not typical of UC, including multiple organized thrombi with recanalization in the veins existing in the submucosal layer to the subserosal layer and an increased infiltration of inflammatory cells. These findings led to the pathological diagnosis of MIVOD.

**Conclusion:**

We report a very rare case in which MIVOD occurred during the course of UC.

## Background

Mesenteric inflammatory veno-occlusive disease (MIVOD) is a rare disease characterized by lymphocytic inflammation of the intestinal wall and mesenteric veins and venules, without arterial involvement and without evidence of systemic vasculitis, which results in intestinal ischemia [1]. MIVOD is difficult to diagnose because of its rarity, nonspecific clinical findings, and frequent confusion with other diseases, along with the necessity for histopathological verification [2]. Drugs, cytomegalovirus, bone marrow transplants, and antiphospholipid antibody syndrome are all involved in the disease, but a detailed pathogenesis remains unknown [3–6]. MIVOD is mostly resistant to medical treatment, as anticoagulants and immunoregulatory drugs have proven ineffective for patients with MIVOD [7, 8]. In most cases, surgical resection of the involved bowel is required and prognosis is typically excellent [7, 8]. Here we report a very rare case in which MOVID occurred during the course of ulcerative colitis (UC).

## Case presentation

**Fig. 1:**
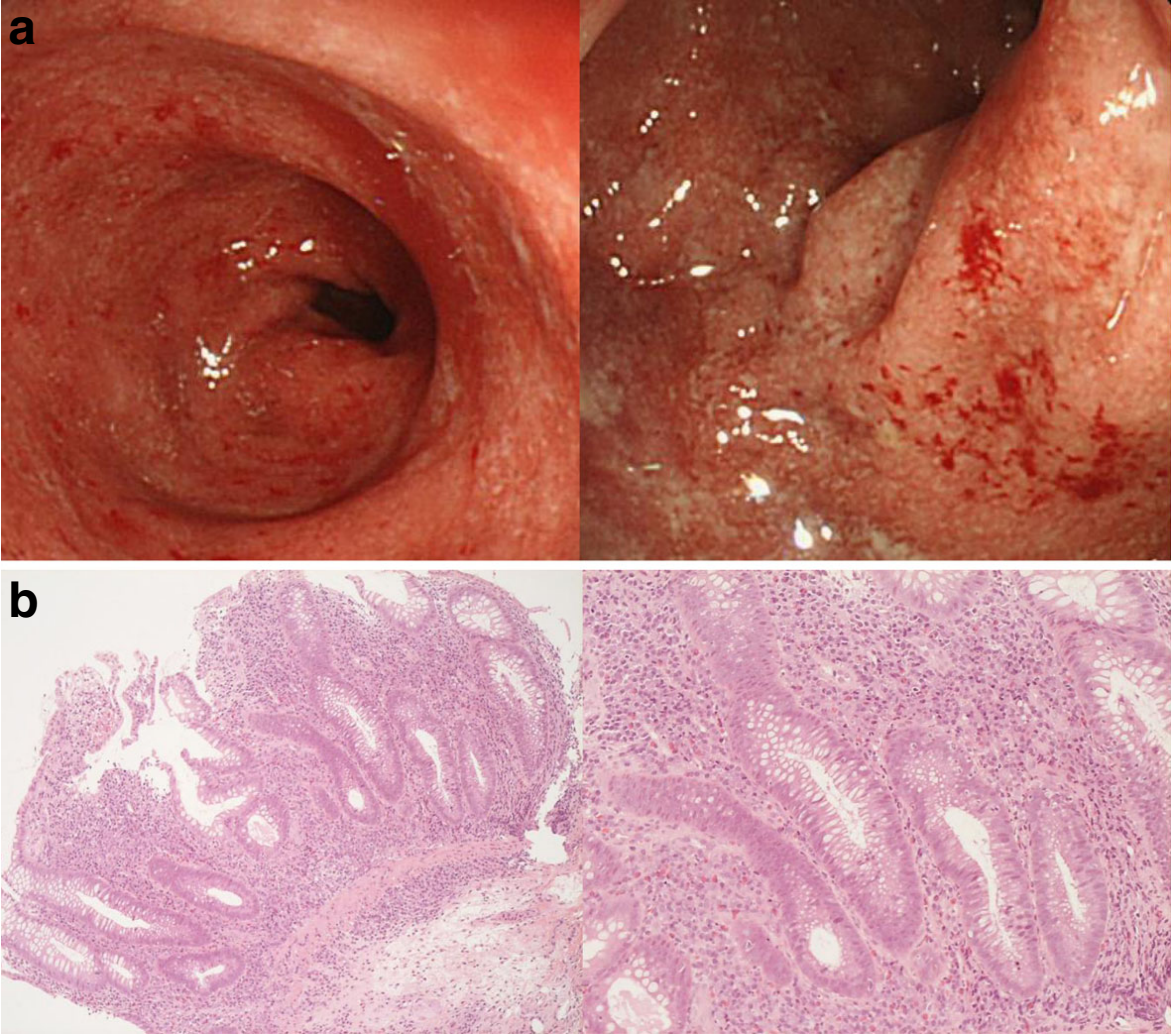
**a** Colonoscopy revealed loss of the vascular appearance, erythema, friability of the mucosa, and spontaneous bleeding in the sigmoid and rectal colon. These endoscopic findings are consistent with active ulcerative colitis. **b** Pathological images showed chronic inflammatory cell infiltration, distortion of crypt architecture, and inflammation of the crypts. Hematoxylin-eosin staining

A 32-year-old man initially visited our hospital with the chief complaint of bloody stool at the age of 29. A colonoscopy performed after hospitalization revealed loss of the vascular appearance, erythema, friability of the mucosa, and spontaneous bleeding in the sigmoid and the rectal colon (Fig. 1a). Histological examination of biopsies showed chronic inflammatory cell infiltration with distortions of crypt architecture and cryptitis (Fig. 1b). Based on these findings, the patient was diagnosed with moderate UC. Although his clinical symptoms were relatively controlled by the oral administration of 5-aminosalicylic acid (5-ASA).

**Fig. 2:**
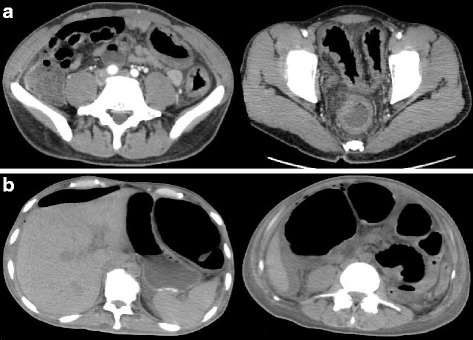
**a** Contrast-enhanced computed tomography showed long segment marked colonic wall thickening extending from the transverse colon to the distal rectum on the first day of hospitalization. **b** Computed tomography revealed abdominal free air and dilated loops of colon on the 35th hospital day

**Fig. 3:**
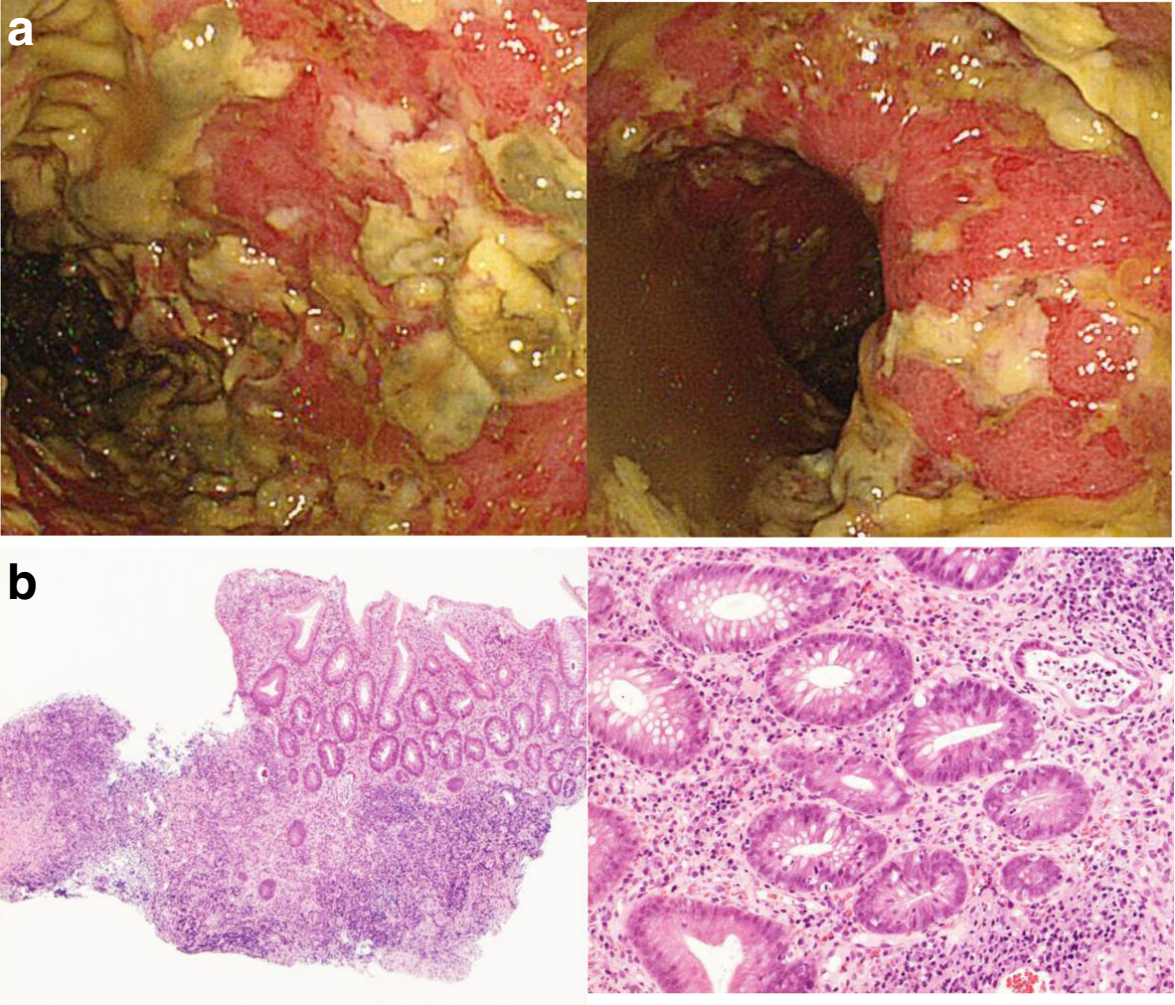
**a** Colonoscopy showed confluent deep ulceration and loss of mucosal architecture. **b** Pathological findings were consistent with possible ulcerative colitis. Hematoxylin-eosin staining

**Fig. 4:**
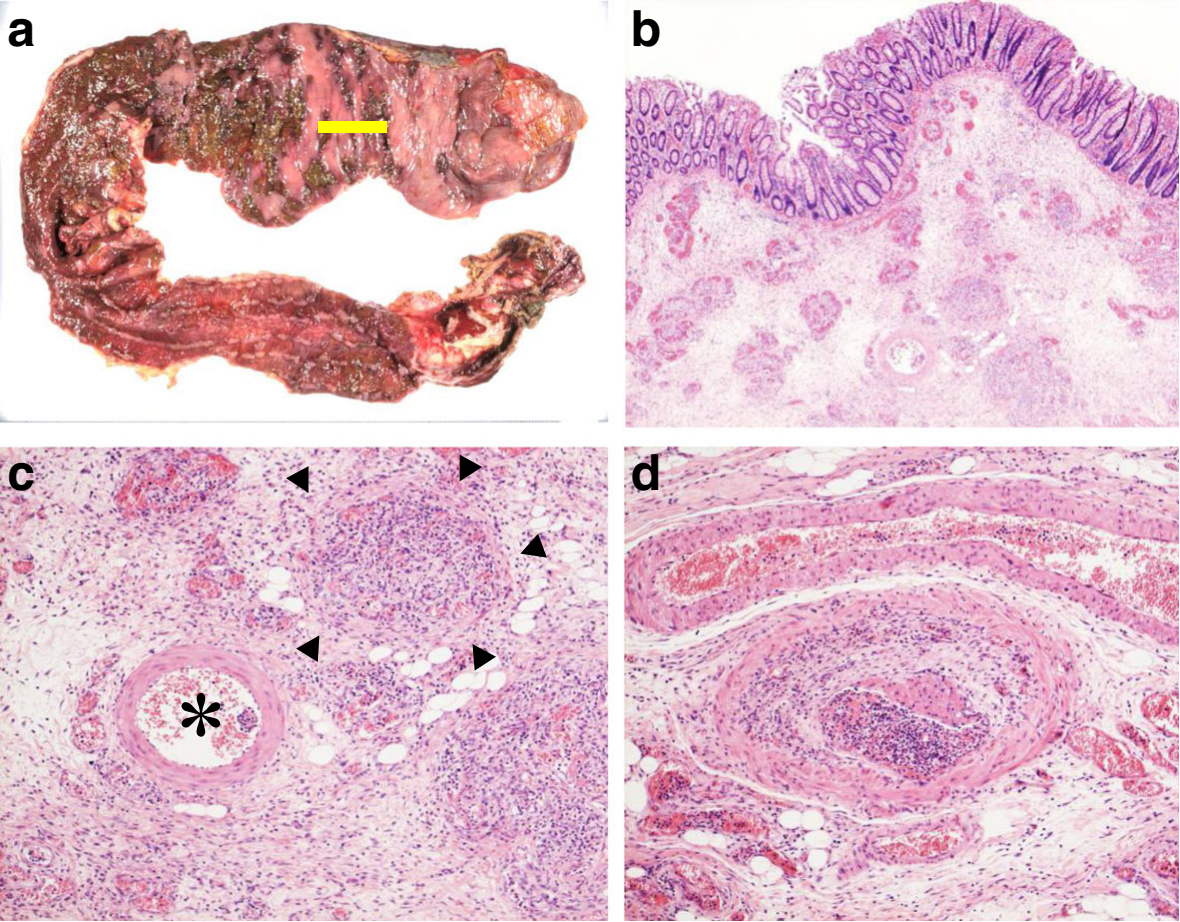
**a** Gross feature of surgical excision specimen. Deep longitudinal ulcers with fusion tendency were scattered in the large intestine. The yellow line indicates the site where the following tissue examination was performed. **b** Histopathology showed only minor changes in the mucosalstructure, but many veins in submucosal layer demonstrated prominent myointimal hyperplasia with narrowed lumens. Hematoxylin-eosin staining. **c** Arteries of submucosal layer were essentially normal (*). Venous thrombi of varying age were identified (arrow heads). **d** Small mesenteric vein and its intramural tributaries showed partly organized thrombi. Hematoxylin-eosin staining

**Fig. 5:**
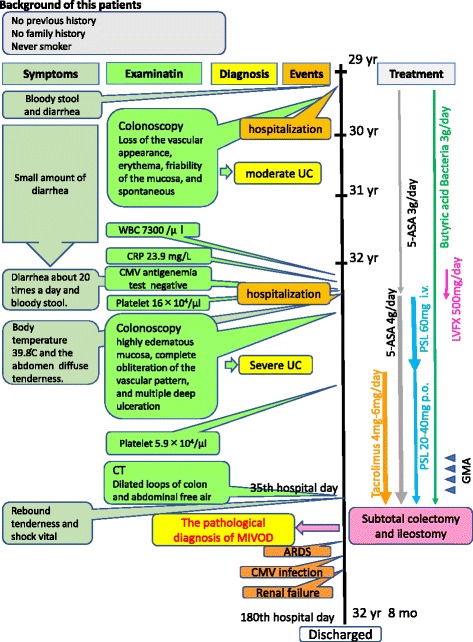
The clinical course of this patient. CT: Computed tomography; CRP: C-reactive protein; CMV: Cytomegalovirus; GMA: Granulocyte Monocyte Apheresis; LVFX: Levofloxacin; MIVOD: Mesenteric inflammatory veno-occlusive disease; PSL: Prednisolone; UC: Ulcerative colitis; 5-ASA: 5-aminosalicylic acid

He was then hospitalized at the age of 32 for severe diarrhea and progressive epigastric pain. He had rectal bleeding and his disease activity was severe according to the Truelove and Witts’ severity index. On physical examination at the time of hospitalization, body temperature was 39.8°C and the abdomen was diffusely tender without muscular guarding or rigidity. Laboratory data, including complete blood count and coagulation profile, were within normal range aside from microcytic and hypochromic anemia with a hemoglobin level of 11.5 g/dL. Serum C-reactive protein (CRP) level was elevated at 23.9 mg/L. Serum tumor markers were within the normal range. Blood, stool, and urine cultures were negative. Cytomegalovirus (CMV) antigenemia test was negative, and antinuclear antibody and serum complement were both in the normal range. Contrast-enhanced computed tomography (CT) showed marked colonic wall thickening extending from the transverse colon to the rectum, but there was no abnormality in the mesenteric vessels and no abdominal free air (Fig. 2a). Colonoscopy revealed highly edematous mucosa, complete obliteration of the vascular pattern, and multiple deep ulcerations extending from the transverse colon to the rectum (Fig. 3a). Histopathology of biopsies obtained during colonoscopy showed findings consistent with possible UC. Basal plasmacytosis and eosinophil infiltration were unremarkable and there was no typical “owl eye” inclusion indicating CMV infection (Fig. 3b). Based on clinical findings, Buerger’s disease, Behçet’s disease, rheumatoid arthritis, and systemic lupus erythematosus were excluded. A diagnosis of severe UC was made and intravenous steroids and antibiotic therapy were started. Since the clinical symptoms and laboratory blood data were not improved by the systemic steroid therapy, the dose of steroids was decreased gradually on the 10th hospital day, while oral administration of tacrolimus was started and maintained at a high trough level. However, his clinical symptoms were completely unresponsive to tacrolimus therapy and surgical colectomy was recommended, but he strongly refused surgery, and consent was not obtained. We performed four sessions of granulocyte and monocyte adsorption apheresis as an additional therapy. On the 35th hospital day, his abdominal pain had worsened with rebound tenderness and ultimately led to a state of shock. CT revealed dilated loops of colon and abdominal free air (Fig. 2b). Following a diagnosis of perforation peritonitis complicated with toxic megacolon, subtotal colectomy and ileostomy were performed (Fig. 4a). Histopathology of the surgically resected colon showed only minor changes in the mucosal histological structure; however, many veins present from the submucosal layer to the subserosal layer demonstrated prominent myointimal hyperplasia with narrowed lumens in contrast to arteries that were essentially normal (Fig. 4b and c). Vasculitis of the small mesenteric veins and their intramural tributaries with thrombosis was also observed (Fig. 4d). These findings confirmed the diagnosis of MIVOD. Despite the occurrence of various postoperative complications, including acute respiratory distress syndrome (ARDS), CMV infection (Given the positive finding of CMV-C7HRP in his blood during management in the postoperative ICU, administration of ganciclovir was performed and we confirmed a negative result for CMV-C7HRP 14 days after administration of ganciclovir.), rectal hemorrhage, and renal failure, the patient gradually improved with multidisciplinary treatment and was discharged after 6 months of hospitalization. He is in remission with oral administration of 5ASA after discharge. The clinical course of this patient is shown in Fig. 5.

## Discussion

The first case of isolated mesenteric venous inflammation and associated thrombosis leading to mesenteric ischemia was described in 1976 [9]. Since then, various terminologies have been used to describe these pathological findings, including necrotizing and enterocolic lymphocytic phlebitis [10], idiopathic myointimal hyperplasia [11], intramural mesenteric venulitis, and most recently, MIVOD.

The clinical symptoms of patients with MIVOD are abdominal pain and mucus per rectum or bloody diarrhea of variable duration. Endoscopic findings are not typical. Several reports show that contrast-enhanced CT and angiography are useful [7, 12]. However, in our case, contrast-enhanced CT showed colonic wall thickening, but was unable to detect the abnormalities of the mesenteric veins. The diagnosis of MIVOD is based on histopathological findings combined with the exclusion of other systemic diseases such as Buerger’s disease, Behçet’s disease, rheumatoid arthritis, and systemic lupus erythematosus [13].

Histopathological findings of MIVOD show isolated vasculitis of the small mesenteric veins and their intramural tributaries with thrombosis as a secondary manifestation, but arterial involvement is not seen [13]. Some subacute cases show granulomatous phlebitis and myointimal hyperplasia, as observed in our case.

In addition to the similarity of the clinical symptoms and endoscopic images between MIVOD and UC, endoscopic biopsies from patients with MIVOD show nonspecific mucosal inflammation consistent with UC because these endoscopic biopsies may not include the submucosal vessels that are necessary for an accurate diagnosis [8]. Therefore, MIVOD is often misdiagnosed and treated as UC, and when the appropriate treatment for UC is unsuccessful, colectomy must be performed, resulting in the pathological diagnosis of MIVOD [14].

Although our case was clinically and pathologically consistent with UC at the disease onset, his clinical course suddenly worsened 3 years later, resulting in a subtotal colectomy and the pathologically proven diagnosis of MIVOD. Because UC and MIVOD have similar endoscopic findings and there are no endoscopic findings specific to MIVOD, it is very difficult to diagnose MIVOD with endoscopic findings alone. In addition, since the postoperative specimen revealed that the surface mucosal structure of the colon at the site where phlebitis and venous thrombus existed was relatively maintained, it seemed unlikely that secondary thrombus was formed by exacerbation of UC. Therefore, we contend that this case of MIVOD developed during the course of UC.

Generally, the postoperative course of MIVOD is good; however, in our case, certain issues remained, such as residual rectal inflammation and persistent small bleeding. Although it is advantageous to remove the residual rectum and close the ileostomy, the possibility of anastomotic leakage must be sufficiently evaluated. At the present time, we currently resort to conservative observation, with the need for careful, continued evaluation of the residual rectum and lower small intestine in the future.

## Conclusion

MIVOD has no specific clinical findings and the final diagnosis is based on the histologic diagnosis of resected intestinal tract, resulting in a small number of reported cases of MIVOD. Furthermore, cases of MIVOD developing during the course of UC appear to be very rare. Further investigation of the mechanism of MIVOD onset and its relationship with UC is necessary through the accumulation of more cases in the future.

## Abbreviations

5-ASA: 5-aminosalicylic acid
ARDS: Acute respiratory distress syndrome
CMV: Cytomegalovirus
CRP: C-reactive protein
CT: Computed tomography
GMA: Granulocyte Monocyte Apheresis
LVFX: Levofloxacin
MIVOD: Mesenteric inflammatory veno-occlusive disease
PSL: Prednisolone
UC: Ulcerative colitis
